# A Coarse-Alignment Method Based on the Optimal-REQUEST Algorithm

**DOI:** 10.3390/s18010239

**Published:** 2018-01-16

**Authors:** Yongyun Zhu, Tao Zhang, Xiang Xu

**Affiliations:** 1 School of Instrument Science and Engineering, Southeast University, Nanjing 210096, China; zhyy@seu.edu.cn (Y.Z.); xuxiang@seu.edu.cn (X.X.); 2 Key Laboratory of Micro-Inertial Instrument and Advanced Navigation Technology, Ministry of Education, Nanjing 210096, China

**Keywords:** strapdown inertial navigation system (SINS), coarse alignment, attitude determination, optimal-REQUEST

## Abstract

In this paper, we proposed a coarse-alignment method for strapdown inertial navigation systems based on attitude determination. The observation vectors, which can be obtained by inertial sensors, usually contain various types of noise, which affects the convergence rate and the accuracy of the coarse alignment. Given this drawback, we studied an attitude-determination method named optimal-REQUEST, which is an optimal method for attitude determination that is based on observation vectors. Compared to the traditional attitude-determination method, the filtering gain of the proposed method is tuned autonomously; thus, the convergence rate of the attitude determination is faster than in the traditional method. Within the proposed method, we developed an iterative method for determining the attitude quaternion. We carried out simulation and turntable tests, which we used to validate the proposed method’s performance. The experiment’s results showed that the convergence rate of the proposed optimal-REQUEST algorithm is faster and that the coarse alignment’s stability is higher. In summary, the proposed method has a high applicability to practical systems.

## 1. Introduction

The strapdown inertial navigation system (SINS) is an autonomous system that calculates the position and orientation of a carrier relative to an initial point and orientation using inertial-sensor measurements [1,2]. The initial attitude, obtained by initial alignment, is therefore significant for the achievement of high-precision navigation. There are two important indexes for the evaluation of initial-alignment performance: the alignment precision and the convergence speed [3,4]. In recent years, many researchers have been devoted to improving the performance of initial alignment, and a series of valuable methods have been proposed [5,6,7].

According to the alignment process, the initial alignment is usually divided into two stages [8,9,10,11]. The first stage is called the coarse-alignment process and is utilized to determine the rough attitude based on the earth’s gravity and angular rate [8,9]. The coarse alignment’s contribution is mainly reflected in the alignment velocity. Therefore, an effective coarse-alignment method can reduce the alignment time so that the system can quickly enter the navigation state. The second stage is the fine-alignment process, which can more accurately determine the initial attitude [10,11]. After the coarse alignment has been conducted, the misalignment angles can converge to a small angle, so that the nonlinear error models of SINS can be approximately simplified into the linear error models. Then, a linear Kalman filter can be applied for the fine alignment. Based on the estimation of the bias of the inertial sensors, the misalignment angles can be further reduced. Consequently, as the coarse alignment is the fine alignment’s premise, its performance will directly affect the fine alignment’s performance. The design of a coarse-alignment algorithm with fast convergence speed and high alignment accuracy is very important to practical applications. This paper will focus on the design of a high-performance coarse-alignment method.

Several methods have been designed to improve coarse-alignment performance; one of them is based on the inertial frame, which can be summed up as the attitude determination (AD) [12]. This means that the calculation of the initial-attitude matrix in [12] is transformed into the determination of the constant direction-cosine matrix (DCM). Generally, one solution to the AD problem focuses on the determination of the attitude matrix [13,14,15]. Qin and Wang [12,14] proposed a method, based on the dual-vector AD, that focused on the coarse alignment on the swing base. However, the method, which was proposed in [12], did not achieve a favorable coarse-alignment performance, as the acceleration of the observation vectors measured by the inertial measurement unit (IMU) contained random noise. Another solution to the AD problem involves the determination of the corresponding attitude quaternion [16,17,18,19,20,21]. In order to solve the initial alignment on the swing base, Zhou [16] proposed a coarse-alignment method based on the quaternion-estimation (QUEST) algorithm, which achieves faster convergence velocity than the dual-vector AD algorithm. However, when determining the attitude quaternion, the QUEST algorithm utilizes only the vector observation obtained at a single time point; the information contained in the past measurements gets lost. Zhu [18] utilized the recursive quaternion estimator (REQUEST) algorithm [19] to achieve the coarse alignment, which determines the attitude quaternion by recursive calculation to make full use of the measurements. Wu [20] proposed an optimization-based alignment (OBA) algorithm for SINS, which establishes the alignment as an optimization problem that involves finding the minimum eigenvector. Compared with the QUEST algorithm, the OBA method achieves a better performance of the coarse alignment. However, the vector observation in the REQUEST algorithm and OBA algorithm also contains random noise. Choukroun [21] proposed an Optimal-REQUEST (OPREQ) algorithm for AD on the basis of the REQUEST algorithm, which adjusts the gain of the filter adaptively to achieve a better performance of AD than the REQUEST algorithm. In order to improve both the convergence velocity and alignment accuracy, this paper proposes a coarse-alignment method based on the optimal-REQUEST (OPREQ) algorithm.

We propose a new inertial-frame-based coarse-alignment method that is inspired by the OPREQ algorithm [21]. Based on the swaying motion’s properties, the coarse alignment of the inertial frame transforms the determination of the initial-attitude matrix into the constant-DCM calculation; we adopted the integral algorithm in order to filter the inertial sensors’ random noise. Then, we utilized the OPREQ algorithm for attitude determination in order to determine the constant DCM. There are two advantages to our proposed coarse-alignment method, which can improve the convergence rate and the stability of the coarse alignment. On the one hand, the proposed method changes the gain of the filter adaptively, rendering the OPREQ filter optimal. On the other hand, the proposed method can filter observation noise by building an accurate measurement model. We verified the performance of this coarse-alignment method with simulation and turntable tests.

The rest of the paper is organized as follows: we introduce the definition of a coordinate frame in the next section. Then, in Section 3, we state the principle of coarse alignment based on the inertial frame. In Section 4, we derive the principle of the OPREQ algorithm. The performance of the proposed method is illustrated through the simulation and turntable tests in Section 5. Finally, our conclusions for this paper are summarized in Section 6.

## 2. Definition of the Coordinate Frame

Some frame definitions in this paper are as follows:

1. *i*-frame: Earth-centered initially-fixed orthogonal reference frame;
2. *n*-frame: orthogonal reference frame aligned with East-North-Up (ENU) geodetic axes;
3. *b*-frame: orthogonal reference frame aligned with IMU axes;
4. *b*0-frame: orthogonal reference frame that is non-rotating relative to the *i*-frame, which is formed by fixing the b-frame at start-up in the inertial space;
5. *e*-frame: Earth-centered Earth-fixed (ECEF) orthogonal reference frame;
6. *e*0-frame: orthogonal reference frame that is non-rotating relative to the *i*-frame, which is formed by fixing the e-frame at start-up in the inertial space.

**Figure 1:**
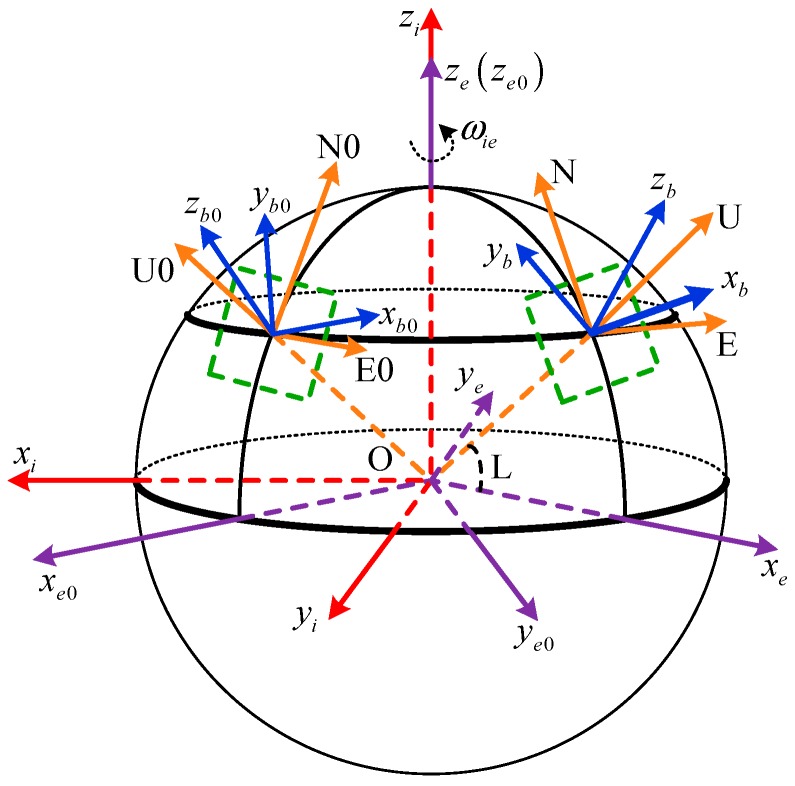
Alignment error curves of three different constant biases.

The above coordinate frames are shown in Figure 1.

## 3. The Principle of Coarse Alignment Based on the Inertial Frame

The attitude matrix can be calculated by the following equation:

(1) $$C_{b}^{n}=C_{e}^{n}C_{e0}^{e}C_{b0}^{e0}C_{b}^{b0}$$

According to the definition of the relevant frame, $C_{e}^{n}$ and $C_{e0}^{e}$ can be calculated as follows:

(2) $$C_{e}^{n}=\left[\begin{array}{ccc} 0 & 1 & 0\\ -sin\left(L\right) & 0 & cos\left(L\right)\\ cos\left(L\right) & 0 & sin\left(L\right) \end{array}\right]$$

(3) $$C_{e0}^{e}=\left[\begin{array}{ccc} cos\left(\omega_{ie}\cdot \left(t-t_{0}\right)\right) & sin\left(\omega_{ie}\cdot \left(t-t_{0}\right)\right) & 0\\ -sin\left(\omega_{ie}\cdot \left(t-t_{0}\right)\right) & cos\left(\omega_{ie}\cdot \left(t-t_{0}\right)\right) & 0\\ 0 & 0 & 1 \end{array}\right]$$

where L denotes the latitude of the carrier and $\omega_{ie}$ denotes the angular rate of the Earth. The attitude transfer matrix $C_{b}^{b0}$ between the *b*- and *b*0-frames can be calculated by the gyroscope output in real time, that is by solving the attitude-matrix differential Equation (4):

(4) $$\{\begin{array}{c} {\dot{C}}_{b}^{b0}=C_{b}^{b0}\left[\omega_{ib}^{b}\times \right]\\ C_{b}^{b0}\left(t_{0}\right)=I_{3} \end{array}$$

Therefore, the key to calculating the attitude matrix $C_{b}^{n}$ is to determine the constant attitude matrix $C_{b0}^{e0}$. Assuming that the carrier does not exhibit a linear motion during the whole alignment process, $f^{b}$ consists of the output of three accelerometers: the gravity-acceleration vector $g^{b}$, the bias of the accelerometer $\nabla^{b}$, and the additional interference acceleration $a^{b}$, which is to say $f^{b}=-g^{b}+\nabla^{b}+a^{b}$. The projection of the vector $f^{b}$ in the *b*0-frame can be calculated as Equation (5):

(5) $$f^{b0}=C_{b}^{b0}f^{b}=C_{b}^{b0}\left(-g^{b}+\nabla^{b}+a^{b}\right)=-C_{e0}^{b0}g^{e0}+C_{b}^{b0}\left(\nabla^{b}+a^{b}\right)$$

where:

(6) $$g^{e0}=C_{e}^{e0}C_{n}^{e}g^{n}=\left[\begin{array}{c} -g\cdot cos\left(L\right)\cdot cos\left(\omega_{ie}\cdot \left(t-t_{0}\right)\right)\\ -g\cdot cos\left(L\right)\cdot sin\left(\omega_{ie}\cdot \left(t-t_{0}\right)\right)\\ -g\cdot sin\left(L\right) \end{array}\right]$$

We used integrals on both sides of Equation (5) to eliminate random noise:

(7) $$\int_{t_{0}}^{t}f^{b0}dt=C_{e0}^{b0}\int_{t_{0}}^{t}-g^{e0}dt+\int_{t_{0}}^{t}C_{b}^{b0}\left(\nabla^{b}+a^{b}\right)dt$$

We denoted $V^{b0}\left(t\right)=\int_{t_{0}}^{t}f^{b0}dt$ and $V^{e0}\left(t\right)=\int_{t_{0}}^{t}-g^{e0}dt$. Ignoring the second integral term on the right, Equation (7) was simplified as:

(8) $$V^{b0}\left(t\right)=C_{e0}^{b0}V^{e0}\left(t\right)$$

where,

(9) $$V^{e0}\left(t\right)=\left[\begin{array}{c} g\cdot cos\left(L\right)\cdot sin\left(\omega_{ie}\cdot \left(t-t_{0}\right)\right)/\omega_{ie}\\ g\cdot cos\left(L\right)\cdot \left[1-cos\left(\omega_{ie}\cdot \left(t-t_{0}\right)\right)\right]/\omega_{ie}\\ g\cdot sin\left(L\right)\cdot \left(t-t_{0}\right) \end{array}\right]$$

We normalized the vectors $V^{b0}\left(t\right)$ and $V^{e0}\left(t\right)$, denoted by b and r:

(10) $$\begin{array}{cc} b=\frac{V^{b0}\left(t\right)}{||V^{b0}\left(t\right)||} & r=\frac{V^{e0}\left(t\right)}{||V^{e0}\left(t\right)||} \end{array}$$

where ||⋅|| denotes the Euclidean norm. Equation (8) can then be rewritten as the vector observations-based measurement model for $C_{e0}^{b0},$ as:

(11) $$b=C_{e0}^{b0}r$$

From Equation (11), we know that the coarse alignment based on the inertial frame can be summed up as an AD problem. Additionally, the DCM matrix $C_{e0}^{b0}$ is a constant attitude matrix. One solution to the AD problem would be to calculate the optimal matrix **A** itself; the other solution involves the determination of the corresponding optimal quaternion ***q***. The three-axis attitude determination (TRIAD) algorithm belongs to the former method. However, the TRIAD algorithm is incapable of achieving an accurate result as it ignores the vector-observation measurement error. The OPREQ algorithm computes the optimal quaternion ***q*** by constructing a matrix K, which can reduce the influence of measurement noise adaptively.

## 4. The Principle of the Optimal-REQUEST Algorithm

Reference [21] expresses that the optimal quaternion ***q*** for the AD problem is the eigenvector of matrix K that belongs to its largest positive eigenvalue. Matrix K can be calculated by Equation (12), given a set of *n* simultaneous observations $b_{i},r_{i},i=1,2,\ldots ,n$, obtained at time $t_{k},$ and the corresponding weights $a_{i}$, where, $\sum_{i=1}^{n}a_{i}=1$. We defined the 4×4 symmetric matrix K as:

(12) $$K_{k/k}=\left[\begin{array}{cc} S_{k}-\sigma_{k}I_{3} & z_{k}\\ z_{k}^{T} & \sigma_{k} \end{array}\right]$$

where the 3×3 matrix $S_{k}$, the 3×1 vector $z_{k}$, and the scalar $\sigma_{k}$ are defined as follows:

(13) $$\begin{array}{cc} B_{k}≜\sum_{i=1}^{n}a_{i}b_{i}r_{i}^{T} & S_{k}≜B_{k}+B_{k}^{T}\\ z_{k}≜\sum_{i=1}^{n}a_{i}b_{i}\times r_{i} & \sigma_{k}≜tr\left(B_{k}\right) \end{array}$$

where tr(⋅) denotes the trace operator. 

The update equation of the attitude quaternion is:

(14) $$q_{k+1}=\Phi_{k}q_{k}$$

where $\Phi_{k}$ is a state transition matrix. If the matrix A is a constant attitude matrix, then q is a constant attitude quaternion and $\Phi_{k}=I_{4}$.

We considered a single observation at time $t_{k+1}$, namely $b_{k+1},r_{k+1}$. The scalar weighting coefficient of the observation at time $t_{k+1}$ was denoted by $a_{k+1}$. The corresponding matrix K at time $t_{k+1}$, denoted by $\delta K_{k+1}$, was constructed as follows:

(15) $$\delta K_{k+1}=\frac{1}{a_{k+1}}\left[\begin{array}{cc} S_{k+1}-\sigma_{k+1}I_{3} & z_{k+1}\\ z_{k+1}^{T} & \sigma_{k+1} \end{array}\right]$$

where the 3×3 matrix $S_{k+1}$, the 3×1 vector $z_{k+1}$, and the scalar $\sigma_{k+1}$ are defined as follows:

(16) $$\begin{array}{cc} B_{k+1}≜a_{k+1}b_{k+1}r_{k+1}^{T} & S_{k+1}≜B_{k+1}+B_{k+1}^{T}\\ z_{k+1}≜a_{k+1}b_{k+1}\times r_{k+1} & \sigma_{k+1}≜tr\left(B_{k+1}\right) \end{array}$$

### 4.1. Measurement Equation

With regards to the observation vectors obtained at time $t_{k+1}$, we supposed that the reference vector $r_{k+1}$ is acquired error-free, while the measurement vector $b_{k+1}$ contains the noise vector $\delta b_{k+1}$. That is:

(17) $$b_{k+1}=A_{k+1}r_{k+1}+\delta b_{k+1}$$

A 4×4 symmetric matrix, which is denoted by $V_{k+1}$, is defined as [19]:

(18) $$V_{k+1}=\frac{1}{a_{k+1}}\left[\begin{array}{cc} S_{b}-\kappa_{b}I_{3} & z_{b}\\ z_{b}^{T} & \kappa_{b} \end{array}\right]$$

where the quantities used in Equation (18) are defined as follows:

(19) $$\begin{array}{cc} B_{b}≜a_{k+1}\delta b_{k+1}r_{k+1}^{T} & S_{b}≜B_{b}+B_{b}^{T}\\ z_{b}≜a_{k+1}\delta b_{k+1}\times r_{k+1} & \kappa_{b}≜tr\left(B_{b}\right) \end{array}$$

Matrix $V_{k+1}$ is the noise matrix contained in the matrix $\delta K_{k+1}$:

(20) $$\delta K_{k+1}=\delta K_{k+1}^{o}+V_{k+1}$$

where $\delta K_{k+1}$ and $\delta K_{k+1}^{o}$ are respectively the noisy and the noise-free matrices of the new vectors obtained at $t_{k+1}$.

### 4.2. Stochastic Models and Measurement Uncertainty

We supposed that the observation vectors $b_{k}$ are symmetrically distributed around their true value. We established an error model to approximately express the mean and covariance of $\delta b_{k}$. The first and the second moments of this model are:

(21) $$E\left[\delta b_{k}\right]=0,\;E\left[\delta b_{k}\delta b_{k+i}^{T}\right]=\mu_{k}\left(I_{3}-b_{k}b_{k+i}^{T}\right)\delta_{k,k+i}$$

for k=1,2,…, where $\mu_{k}$ is the variance of the component of $\delta b_{k}$.

We noticed that $V_{k}$ is a linear function of $\delta b_{k}$ and $r_{k}$. As $\delta b_{k}$ is a zero-mean white-noise process, $V_{k}$ is also a zero-mean white-noise process. Thus:

(22) $$E\left[V_{k}V_{k+i}^{T}\right]=0_{4}$$

where i≠0. For the zero-mean matrix $V_{k}$, the measurement uncertainty ${ℛ}_{k}$ is defined as:

(23) $${ℛ}_{k}≜E\left[V_{k}V_{k}^{T}\right]$$

We divided the 4×4 matrix ${ℛ}_{k}$ into four parts:

(24) $${ℛ}_{k}=\left[\begin{array}{cc} {ℛ}_{11} & {ℛ}_{12}\\ {ℛ}_{21} & {ℛ}_{22} \end{array}\right]$$

where ${ℛ}_{11}$ is a 3×3 submatrix. The expressions ${ℛ}_{11}$, ${ℛ}_{12}$, ${ℛ}_{21},$ and ${ℛ}_{22}$ were calculated as follows:

(25) $$\begin{array}{c} {ℛ}_{11}=\mu_{k}/n_{k}\left\{\overset{n_{k}}{\underset{i=1}{\sum }}\left[3-{\left(r_{i}^{T}b_{i}\right)}^{2}\right]I_{3}+\left(b_{i}^{T}r_{i}\right)\left(b_{i}r_{i}^{T}+r_{i}b_{i}^{T}\right)+\left(r_{i}\times \right)\left(b_{i}b_{i}^{T}\right){\left(r_{i}\times \right)}^{T}\right\}\\ \begin{array}{c} {ℛ}_{12}=0\\ {ℛ}_{21}=0^{T}\\ {ℛ}_{22}=2\mu_{k}/n_{k} \end{array} \end{array}$$

We supposed that the set of $n_{k}$ simultaneous observations obtained at time $t_{k}$ have the same variance $\mu_{k}$. Additionally, $\sum_{i=1}^{n_{k}}a_{i}=1$. The detailed calculation of ${ℛ}_{k}$ is provided in Reference [21].

### 4.3. Measurement Update Process

Through the above analysis, we obtained the predicted estimate matrix $K_{k/k}$ and the new observation matrix $\delta K_{k+1}$. We then utilized an iterative calculation method to calculate the updated estimate matrix $K_{k+1/k+1}$. The updated estimate $K_{k+1/k+1}$ is calculated via the convex combination of the priori estimate $K_{k/k}$ and the new observation $\delta K_{k+1}$, that is:

(26) $$K_{k+1/k+1}=\left(1-\rho_{k+1}\right)\frac{m_{k}}{m_{k+1}}K_{k/k}+\rho_{k+1}\frac{\delta m_{k+1}}{m_{k+1}}\delta K_{k+1}$$

where $\delta m_{k+1}$ is a positive scalar weight and $m_{k+1}$ is computed as follows:

(27) $$m_{k+1}=\left(1-\rho_{k+1}\right)m_{k}+\rho_{k+1}\delta m_{k+1}$$

for k=0,1,… and $m_{0}=\delta m_{0}$. The scalar $\rho_{k+1}\in (0,1)$ is the gain of Equation (26). If the value of the scalar $\rho_{k+1}$ is fixed, the algorithm is called a REQUEST algorithm [19]. The determination of the scalar $\rho_{k+1}$ is tentative, rendering the REQUEST algorithm suboptimal. We wished to find the optimal value of the scalar $\rho_{k+1}$, which could be changed adaptively according to the estimated residual. In doing so, the convergence of the attitude determination would be faster and the result would become more accurate.

The estimate errors of the algorithm are defined as follows:

(28) $$\begin{array}{c} \Delta K_{k/k}≜K_{k/k}^{o}-K_{k/k}\\ \Delta K_{k+1/k+1}≜K_{k+1/k+1}^{o}-K_{k+1/k+1} \end{array}$$

where $\Delta K_{k/k}$ and $\Delta K_{k+1/k+1}$ respectively represent the priori and posteriori estimation errors.

We supposed that the priori estimate $K_{k/k}$ is unbiased, that is to say $E\left[\Delta K_{k/k}\right]=0$. The expression $K_{k+1/k+1}^{o}$ was calculated by:

(29) $$K_{k+1/k+1}^{o}=\left(1-\rho_{k+1}\right)\frac{m_{k}}{m_{k+1}}K_{k/k}^{o}+\rho_{k+1}\frac{\delta m_{k+1}}{m_{k+1}}\delta K_{k+1}^{o}$$

By subtracting Equation (26) from Equation (29), we obtained the relation between the priori and posteriori errors:

(30) $$\Delta K_{k+1/k+1}=\left(1-\rho_{k+1}\right)\frac{m_{k}}{m_{k+1}}\Delta K_{k/k}+\rho_{k+1}\frac{\delta m_{k+1}}{m_{k+1}}V_{k+1}$$

where $V_{k+1}$ is the measurement error defined in Equation (19). Taking the expectation of both sides of Equation (30), we obtained:

(31) $$E\left[\Delta K_{k+1/k+1}\right]=\left(1-\rho_{k+1}\right)\frac{m_{k}}{m_{k+1}}E\left[\Delta K_{k/k}\right]+\rho_{k+1}\frac{\delta m_{k+1}}{m_{k+1}}E\left[V_{k+1}\right]$$

As mentioned above, the priori error $\Delta K_{k/k}$ and the measurement error $V_{k+1}$ are zero-mean. We know from Equation (30) that $\Delta K_{k+1/k+1}$ is the linear function of $\Delta K_{k/k}$ and $V_{k+1}$. Therefore, the posteriori error is zero-mean. 

The measurement uncertainty corresponding to the two estimate errors is defined as follows:

(32) $$\begin{array}{c} P_{k/k}≜E\left[\Delta K_{k/k}\Delta K_{k/k}^{T}\right]\\ P_{k+1/k+1}≜E\left[\Delta K_{k+1/k+1}\Delta K_{k+1/k+1}^{T}\right] \end{array}$$

According to Equation (30), it can be calculated as the following expression:

(33) $$\begin{array}{l} \Delta K_{k+1/k+1}\Delta K_{k+1/k+1}^{T}\\ \;={\left[\left(1-\rho_{k+1}\right)\frac{m_{k}}{m_{k+1}}\right]}^{2}\times \Delta K_{k/k}\Delta K_{k/k}^{T}-\left(1-\rho_{k+1}\right)\rho_{k+1}\frac{m_{k}\delta m_{k+1}}{m_{k+1}^{2}}\\ \;\times \left(\Delta K_{k/k}V_{k+1}^{T}+V_{k+1}\Delta K_{k/k}^{T}\right)+{\left[\rho_{k+1}\frac{\delta m_{k+1}}{m_{k+1}}\right]}^{2}V_{k+1}V_{k+1}^{T} \end{array}$$

As the priori error $\Delta K_{k/k}$ only contains the observations from time $t_{0}$ to $t_{k}$, $\Delta K_{k/k}$ and $V_{k+1}$ are uncorrelated. Thus:

(34) $$E\left[\Delta K_{k/k}V_{k+1}^{T}\right]=E\left[V_{k+1}\Delta K_{k/k}^{T}\right]=0_{4}$$

Taking the expectation of both sides of Equation (33), we obtained:

(35) $$P_{k+1/k+1}={\left[\left(1-\rho_{k+1}\right)\frac{m_{k}}{m_{k+1}}\right]}^{2}E\left[\Delta K_{k/k}\Delta K_{k/k}^{T}\right]+{\left[\rho_{k+1}\frac{\delta m_{k+1}}{m_{k+1}}\right]}^{2}E\left[V_{k}V_{k}^{T}\right]$$

We used $P_{k/k}$ and ${ℛ}_{k+1}$ to denote, respectively, the first and the second terms of the right-hand side of Equation (35).

(36) $$P_{k+1/k+1}={\left[\left(1-\rho_{k+1}\right)\frac{m_{k}}{m_{k+1}}\right]}^{2}P_{k/k}+{\left[\rho_{k+1}\frac{\delta m_{k+1}}{m_{k+1}}\right]}^{2}{ℛ}_{k+1}$$

Equation (36) represents the uncertainty update in the K-matrix estimation process for any $\rho_{k+1}$, when a new measurement is acquired.

### 4.4. Optimal Gain

We wanted the estimation uncertainty to decrease as much as possible when a new measurement was acquired. Reference [21] proposed that the trace of the matrix P expresses the measurement uncertainty in a way that is suitable. Here, a cost function is defined as:

(37) $$J_{k+1}≜tr\left(E\left[\Delta K_{k+1/k+1}\Delta K_{k+1/k+1}^{T}\right]\right)=tr\left(P_{k+1/k+1}\right)$$

Hence, the design problem of the filter gain $\rho_{k+1}$ comes down to solving the following minimization problem with respect to $\rho_{k+1}$:

(38) $$\underset{\rho_{k+1}\in \left(0,1\right)}{\min}tr\left(P_{k+1/k+1}\right)$$

By substituting Equation (36) into Equation (37), we obtained:

(39) $$J_{k+1}\left(\rho_{k+1}\right)={\left[\left(1-\rho_{k+1}\right)\frac{m_{k}}{m_{k+1}}\right]}^{2}tr\left(P_{k/k}\right)+{\left[\rho_{k+1}\frac{\delta m_{k+1}}{m_{k+1}}\right]}^{2}tr\left({ℛ}_{k+1}\right)$$

The first-order necessary condition for an extremum of $J_{k+1}$ is:

(40) $$\frac{dJ_{k+1}}{d\rho_{k+1}}=2\left[{\left(\frac{m_{k}}{m_{k+1}}\right)}^{2}tr\left(P_{k/k}\right)+{\left(\frac{\delta m_{k+1}}{m_{k+1}}\right)}^{2}tr\left({ℛ}_{k+1}\right)\right]\rho_{k+1}-2{\left(\frac{m_{k}}{m_{k+1}}\right)}^{2}tr\left(P_{k/k}\right)=0$$

As a result, in order for $\rho_{k+1}^{*}$ to yield a stationary point for $J_{k+1},$ the following condition must apply:

(41) $$\rho_{k+1}^{*}=\frac{m_{k}^{2}tr\left(P_{k/k}\right)}{m_{k}^{2}tr\left(P_{k/k}\right)+\delta m_{k+1}^{2}tr\left({ℛ}_{k+1}\right)}$$

It can be shown that Equation (41) is the sufficient condition for the minimum value of the cost function $J_{k+1}$. When the priori estimate’s uncertainty is above the measurement uncertainty, the gain approaches 1 and assigns a high weight to the new update-stage measurement described in Equation (26). On the other hand, when the measurement uncertainty is higher than the priori-estimate uncertainty, the gain approaches 0, and the filter assigns a low weight to the new measurement.

The OPREQ algorithm presented in this section is summarized in Table 1:

## 5. Experimental Analysis

**Figure 2:**
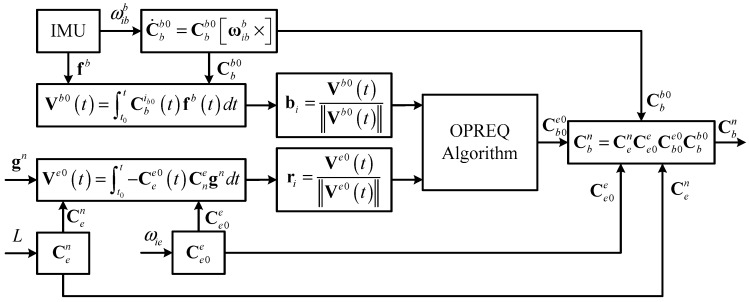
The alignment procedure of the proposed OPREQ algorithm.

Based on the aforementioned analysis, we described the detailed experimental process as follows. First, the constant DCM matrix $C_{e0}^{b0}$ for the coarse alignment of the inertial frame can be selected as the sought attitude matrix A. The vectors $V^{b0}\left(t_{k}\right)$ and $V^{e0}\left(t_{k}\right),$ computed at every update period, are used to construct the measurement vector $b_{k}$ and reference vector $r_{k}$, respectively. Since the measurement vector $b_{k}$ and reference vector $r_{k}$ obtained at any given time always constitute a single observation, the parameter $n_{k}$ and the corresponding weight $a_{k}$ are both equal to 1. Following this, the optimal quaternion q corresponding to the DCM matrix $C_{e0}^{b0}$ is calculated via the OPREQ algorithm. Figure 2 summarizes the alignment procedure of the proposed algorithm.

### 5.1. Simulation Test for Attitude Determination

**Figure 3:**
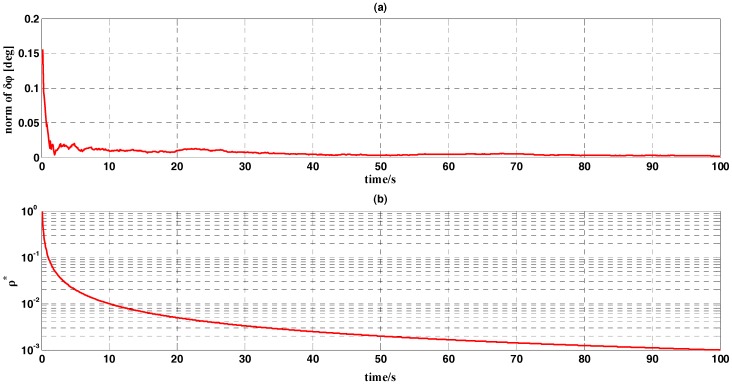
The simulation results of attitude determination based on the OPREQ algorithm. (**a**) the curve of the norm of δϕ; (**b**) the curve of the gain $\rho^{*}$.

**Figure 4:**
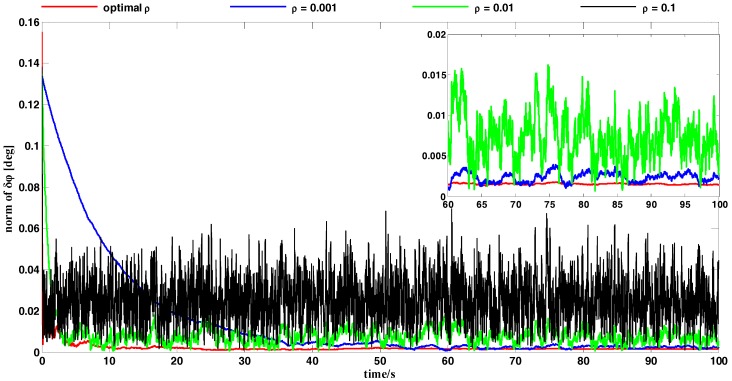
A comparison of the OPREQ and REQUEST algorithms.

In this subsection, we conducted a simulation test for attitude determination on measurement noise in order to verify the effectiveness of the OPREQ algorithm. We assumed that the body frame was static relative to the inertial reference system. The reference vectors we acquired were error-free, while the noise contained in the measurement vectors was modeled as a zero-mean white-noise with an angular standard deviation of 0.1 degrees. Since the body frame was fixed with respect to the reference frame, the gyro output used to measure the angular velocity between the body and reference frames only included the bias. The noise contained in the gyroscope was modeled as a zero-mean Gaussian white-noise with a standard deviation of $0.1^{{}^{\circ}}/h$. The sample rates of the vector observation and gyroscope were both 100 Hz, and different single-vector observations were obtained at each sampling time. The whole simulation test lasted for 100 s. The results are shown in Figure 3 and Figure 4.

Figure 3a shows the curve of the attitude error δϕ′s norm, calculated by the optimal REQUEST algorithm. The figure shows that the norm is approximately steady at 0.003 degrees. Figure 3b represents the curve of the optimal gain $\rho^{*}$ during the whole simulation process. The optimal gain drops gradually from the initial value 1 to 0.001. Figure 3b shows that at the beginning of the estimation process, the optimal REQUEST algorithm puts more weight on the incoming observation. As the number of managed observations increases, the algorithm puts less weight on the new observation. Through the OPREQ algorithm, the estimated attitude approaches the real attitude successfully.

Figure 4 compares the attitude error between the OPREQ algorithm and several different cases of the REQUEST algorithm. We chose three kinds of constant values for gain: 0.001, 0.01, and 0.1. These are typical values within the range of the optimal gain $\rho^{*}$ as shown in Figure 3b. Figure 4 clearly shows that the red line representing the OPREQ algorithm has the fastest convergence speed and the highest accuracy. Out of the three kinds of REQUEST algorithms, the black line representing ρ=0.1 converges fastest because of the measurements’ relatively large weight. Nevertheless, its error still has a relatively high steady state ($0.025^{{}^{\circ}}$) and shows random variations. For ρ=0.1, represented by the blue line, the algorithm converges smoothly and steadily reaches a value of $0.003^{{}^{\circ}}$. Meanwhile, a very low gain puts very little weight on the measurements so that the algorithm has a very slow convergence velocity. Therefore, the OPREQ algorithm can quickly and smoothly achieve the error convergence because the algorithm’s gain changes adaptively.

### 5.2. Simulation Test for the Coarse Alignment

In order to verify the effectiveness of the proposed algorithm for the coarse alignment on the swing base, we used the sinusoidal motion model in order to simulate the IMU swing motion in a ship. The swing model was set as Asin(2πft+φ)+θ, where the parameters A and f are the amplitude and frequency of the swing motion, and the quantities φ and θ represent, respectively, the initial phase and swaying center. The parameters of this swing model are listed in Table 2.

**Figure 5:**
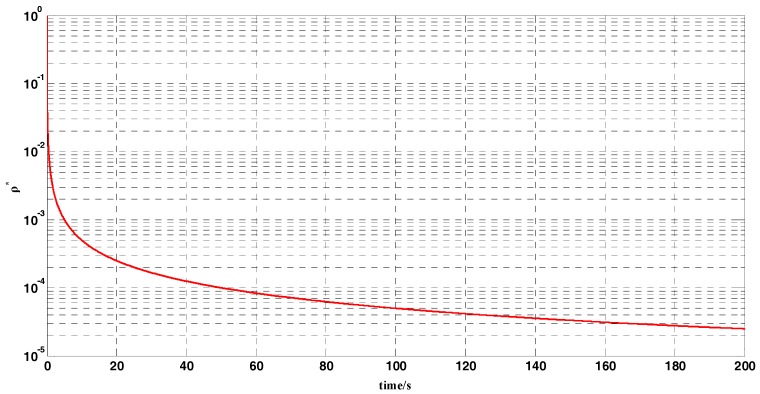
The optimal-gain-$\rho^{*}\;\mathrm{curve}$.

**Figure 6:**
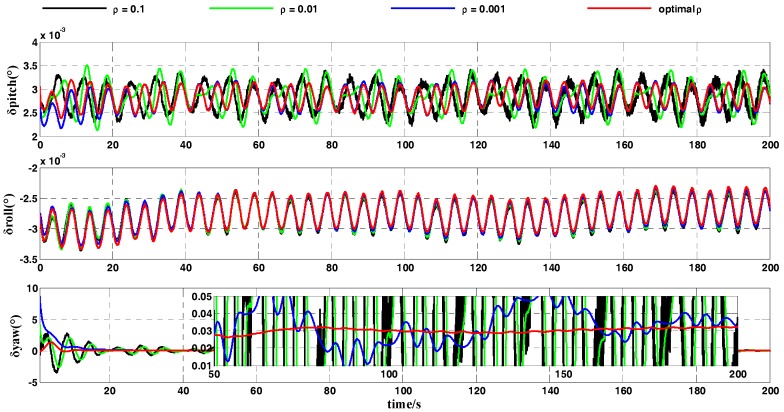
The comparison of the attitude errors between OPREQ and REQUEST.

**Figure 7:**
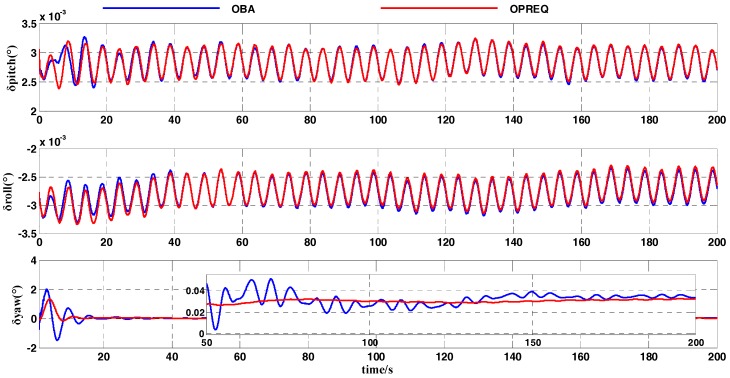
Comparison of attitude errors between the OPREQ and OBA algorithms.

In a practical inertial navigation system, several errors occur in the output of gyroscopes and accelerometers. We assumed that inertial-sensor outputs contain both constant and random errors. In the simulation test, the error parameters of the gyroscopes and accelerometers were set as shown in Table 3. As the carrier does not move in the swing motion, the position of the carrier was fixed in the simulation test. The geographic latitude and longitude of the carrier were set to $L=32^{{}^{\circ}}\left(N\right)$ and $\lambda =118^{{}^{\circ}}\left(E\right)$, respectively. The whole coarse alignment of this test lasted for 200 s. The curve of the optimal gain $\rho^{*}$ is displayed in Figure 5. Figure 6 and Figure 7 show the attitude-error curves of the simulation test. The statistics of each algorithm’s attitude errors are shown in Table 4 and Table 5.

Figure 5 shows the curve of the optimal gain $\rho^{*}$ during the whole coarse-alignment test with the OPREQ algorithm. The optimal gain decreases gradually from the initial value 1 to 0.001 in 200 s. In this coarse-alignment simulation test, we chose, for comparison, three constant values for the gain of the REQUEST algorithm: 0.1, 0.01, and 0.001.

Figure 6 contrasts the attitude errors of the OPREQ algorithm and several REQUEST algorithms. The three subgraphs in Figure 6 represent the error of the pitch angle, the error of the roll angle, and the error of the heading angle. Figure 6 clearly shows that the attitude errors of the horizontal angle are very similar for several methods, approaching the accuracy limit. Several methods’ differences are mainly reflected in the heading-angle error. The heading-angle error in Figure 6 shows that the blue curve representing ρ=0.001 converges slowly at the beginning of the simulation but that the error curve is relatively smooth. While the convergence speed of the black curve (ρ=0.1) is faster, the curve’s amplitude is larger. The red curve is of the OPREQ algorithm. The value of the gain in the OPREQ algorithm changes adaptively; this method not only exhibits the fastest convergence speed in the initial stage but also reaches, with stability, the highest accuracy in the final phase. In order to clearly compare the simulation results of several algorithms, we calculated the mean and variance of several attitude errors in the first 100 s and in the second 100 s. The statistic results of the simulation test are shown in Table 4.

Table 4 clearly shows that the horizontal-angle errors of several algorithms all approach the accuracy limit ($0.0028^{{}^{\circ}}$ and $-0.0028^{{}^{\circ}}$), while the mean and standard deviation of the heading-angle errors among these methods are markedly different. The mean of the heading-angle error in the first 100 s is (optimal ρ)<(ρ=0.1)<(ρ=0.01)<(ρ=0.001), while the standard deviation of the heading-angle error in the last 100 s is (optimal ρ)<(ρ=0.001)<(ρ=0.01)<(ρ=0.1). Among the three REQUEST algorithms, the mean of the heading-angle error for ρ=0.1 is the smallest. That is because the filter puts a relatively large amount of weight on the new measurement, so that this method converges fast at the beginning of the coarse alignment. The standard deviation of the heading-angle error for ρ=0.001 is the most optimal because a very low weight is given to the new measurement. The OPREQ algorithm combines the advantages of several REQUEST methods, so that the mean and the variance of the heading-angle error are inferior to those of the REQUEST algorithm for the whole alignment process.

In this simulation test, we compared the OPREQ algorithm with the OBA algorithm in order to verify the effectiveness of the former method in the coarse alignment. The heading-angle error in Figure 7 shows that the convergence speed of the OPREQ algorithm is faster than that of the OBA algorithm. Additionally, the heading-angle error of the OBA algorithm exhibits a relatively large amplitude. In other words, the result of the OPREQ algorithm is more stable than that of the OBA algorithm. This therefore confirms the advantages of the OPREQ algorithm for both convergence speed and alignment stability. Table 5 shows the error statistics of the two algorithms.

As Table 5 clearly shows, the horizontal-angle errors of the two algorithms are relatively similar; the emphasis should be placed on comparing the heading-angle errors. The standard deviation of the OPREQ algorithm’s heading-angle error is inferior to that of the OBA algorithm for the whole alignment process, which proves the OPREQ algorithm’s stability. Additionally, the alignment accuracy of the OPREQ algorithm is higher than that of the OBA algorithm, because the former has a smaller heading-angle error between 101 and 200 s.

### 5.3. Turntable Test

**Figure 8:**
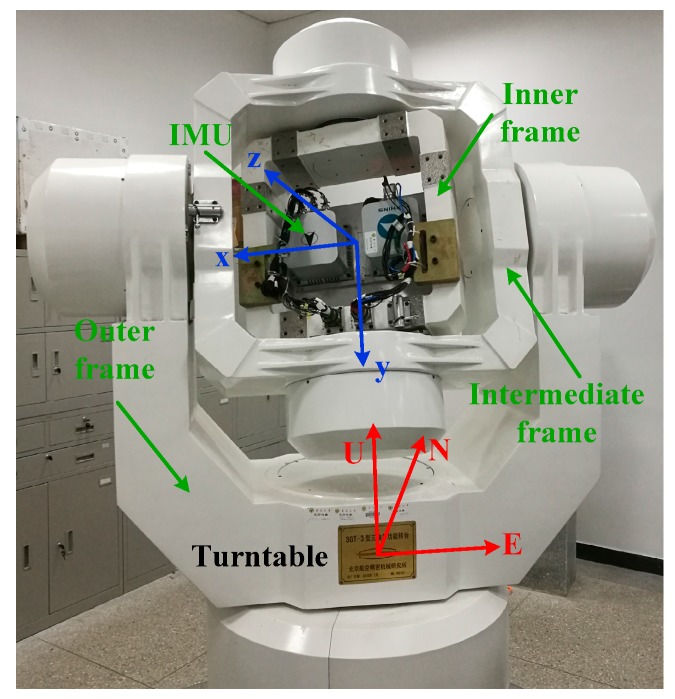
The turntable.

This subsection delineates how we used the turntable test on the swing base in order to verify how feasible and reliable the OPREQ algorithm is in practical environments. We installed the equipment in the turntable as shown in Figure 8. The three-axis turntable can achieve an angle precision of $\pm 0.0001^{{}^{\circ}}$, and the accuracy of the angular velocity is $\pm 0.0005^{{}^{\circ}}/s$. In this test, we used the turntable output’s angle data as the attitude reference because of the turntable’s high angle accuracy. We regarded the outputs of the inner, intermediate, and outer frames as the vehicle’s reference angles in pitch, roll, and yaw, respectively. The IMU used in the turntable test is an Optical fiber SINS, named FOSN, produced by Chinese CASIC hospitals 33. The IMU was installed in the center of the turntable. As Figure 8 shows, the IMU was mounted on a plate in the turntable’s inner frame. The *n*-frame of the turntable was fixed, and the *b*-frame of the IMU changed with the rotation of the turntable. We calculated and compensated both the installing and system errors prior to the turntable test. The IMU we used in this test contained three mutually-orthogonal fiber-optic gyroscopes and three mutually-orthogonal quartz accelerometers. The inertial sensors’ parameters are listed in Table 6.

**Figure 9:**
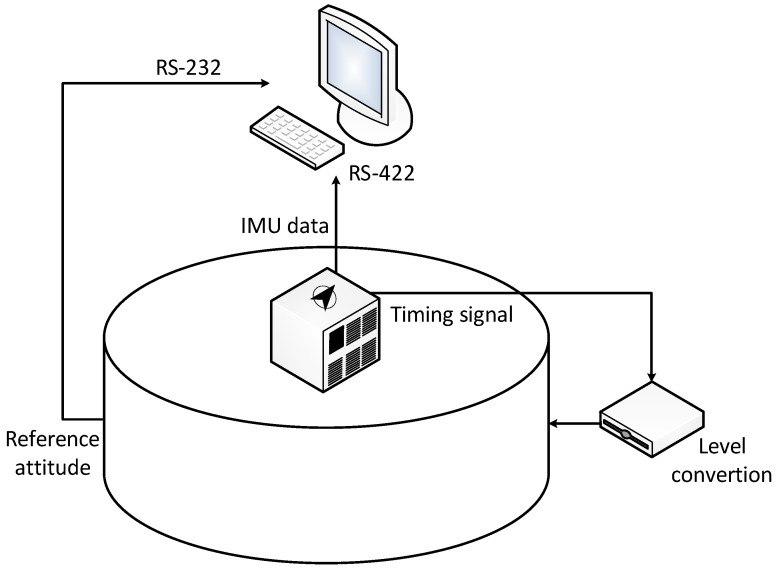
Structure of the turntable test.

Figure 9 shows the structure of the turntable test. On the one hand, the IMU outputs are sent to the navigation computer via the serial port at a frequency of 200 Hz. On the other hand, the time-synchronization signal sent by the IMU causes the turntable to transmit the reference attitude to the navigation computer via a level conversion. The navigation computer saves the outputs of both the IMU and the turntable simultaneously and utilizes the original data to obtain the coarse alignment according to the proposed algorithm.

**Figure 10:**
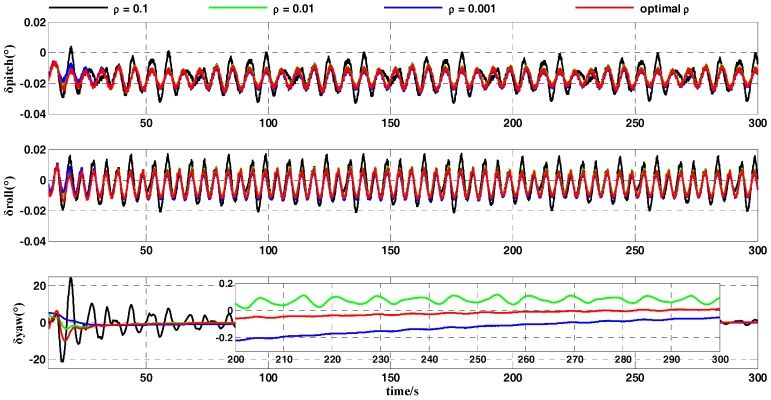
Alignment-error curves of the REQUEST and OPREQ algorithms.

**Figure 11:**
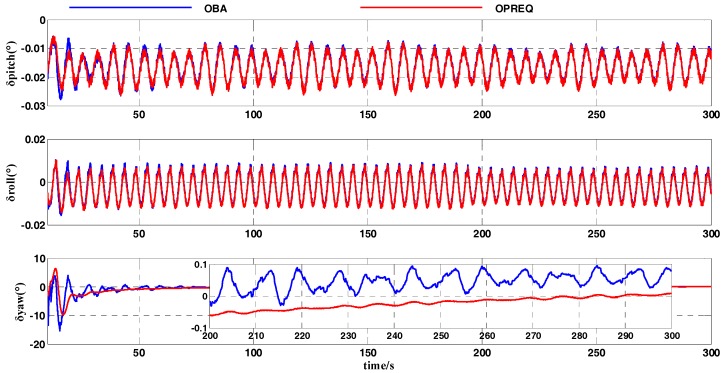
Alignment-error curves of the OBA and OPREQ algorithms.

The IMU motion parameters that we set for the turntable test are shown in Table 7. The geographic latitude and longitude of the turntable were set to $L=32.057^{{}^{\circ}}\left(N\right)$ and $\lambda =118.786^{{}^{\circ}}\left(E\right)$, respectively. The whole coarse alignment of this turntable test lasted for 300 s. The results of the turntable test are shown in Figure 10 and Figure 11. Figure 10 compares the alignment errors of several REQUEST methods with the OPREQ algorithms, including the pitch angle, roll angle, and yaw angle. Meanwhile, the mean and standard deviations of the attitude errors in the different alignment stages were calculated as shown in Table 8. Additionally, Figure 11 shows a comparison of the attitude-error curves of the OBA and OPREQ algorithms, and Table 9 lists the statistical data of the attitude errors for those two algorithms.

Figure 10 shows that the OPREQ algorithm’s convergence speed is faster than several REQUEST algorithms and that its alignment accuracy is the most optimal. In addition, the error statistics in Table 8, especially those in the later stage of the alignment process, show that the mean and standard deviations of the attitude errors for the OPREQ algorithm are inferior to those of any other algorithms.

Figure 11 shows that the OPREQ algorithm’s convergence velocity is faster than that of the OBA algorithm and that its alignment accuracy is also higher. The error statistics shown in Table 9 further confirm the validity of the OPREQ algorithm. The result of the turntable test shows that the OPREQ algorithm performs well in a practical system.

## 6. Conclusions

In this paper, we proposed, based on the OPREQ algorithm, a coarse-alignment method for the inertial frame. We began by introducing the principle of the coarse alignment of the inertial frame. Then, we deduced the OPREQ algorithm for the attitude determination, which can adaptively change the gain in order to filter the observation noise. Given that it was possible to sum up the coarse alignment of the inertial frame as the solution of a constant-attitude matrix, we were able to utilize the OPREQ algorithm to solve the coarse alignment on the swing base. Finally, we carried out simulation and physical experiments in order to verify how the proposed algorithms performed. The results of the corresponding tests showed that the convergence velocity and alignment accuracy of the proposed algorithm are better than those of the OBA and REQUEST algorithms. The method presented in this paper has a particularly high application value for the coarse alignment on the swing base. In a future study, we will aim to apply this method to the coarse alignment on the moving base.

## Figures and Tables

**Table 1:** Attitude determination based on the OPREQ algorithm.

Initialization:	Set $K_{0/0}=\delta K_{0}$, $P_{0/0}={ℛ}_{0}$, $a_{i}=1$, $\rho_{0}^{*}=1$, and $m_{0}=\delta m_{0}=1$.
Step 1:	Set k=k+1;
Step 2:	Compute the matrix $K_{k/k}$ according to Equation (12), compute the matrix $\delta K_{k+1}$ according to Equation (15);
Step 3:	Compute the matrix ${ℛ}_{k+1}$ according to Equations (24) and (25);
Step 4:	Update the gain $\rho_{k+1}^{*}$ by equation (41);
Step 5:	Compute the factor $m_{k+1}$ according to Equation (27);
Step 6:	Update the matrix $K_{k+1/k+1}$ according to Equation (26);
Step 7:	Compute the attitude quaternion $q_{k+1}$ at the current time;
Step 8:	Compute the matrix $P_{k+1/k+1}$ according to Equation (36);
Step 9:	Go to Step 1 until the end.

**Table 2:** The parameters of the swing model.

Items	Pitch (θ)	Roll (γ)	Yaw (ψ)
Amplitude (°)	8	10	6
Frequency (Hz)	0.15	0.125	0.2
Initial phase (°)	0	0	0
Swaying center (°)	0	0	0

**Table 3:** The inertial measurement unit (IMU) parameters.

Parameters		*x*-Axis	*y*-Axis	*z*-Axis
Gyroscope	Constant bias (°/h)	0.01	0.01	0.01
	Random bias (°/h)	0.01	0.01	0.01
	Update frequency (Hz)	200	200	200
Accelerometer	Constant bias (μg)	50	50	50
	Random bias (μg)	50	50	50
	Update frequency (Hz)	200	200	200

**Table 4:** The error statistics of the OPREQ and REQUEST algorithms.

Items			ρ=0.1	ρ=0.01	ρ=0.001	Optimal ρ
Pitch (°)	1–100 s	Mean	2.8338 × 10^−3^	2.8297 × 10^−3^	2.7804 × 10^−3^	2.8185 × 10^−3^
		STD	2.8364 × 10^−4^	3.1746 × 10^−4^	2.4115 × 10^−4^	2.1242 × 10^−4^
	101–200 s	Mean	2.8293 × 10^−3^	2.8277 × 10^−3^	2.8275 × 10^−3^	2.8514 × 10^−3^
		STD	2.9023 × 10^−4^	3.0733 × 10^−4^	2.1463 × 10^−4^	2.0566 × 10^−4^
Roll (°)	1–100 s	Mean	−2.8081 × 10^−3^	−2.7974 × 10^−3^	−2.7896 × 10^−3^	−2.8051 × 10^−3^
		STD	2.3238 × 10^−4^	2.3528 × 10^−4^	2.3058 × 10^−4^	2.4626 × 10^−4^
	101–200 s	Mean	−2.7770 × 10^−3^	−2.7665 × 10^−3^	−2.7594 × 10^−3^	−2.7169 × 10^−3^
		STD	2.2319 × 10^−4^	2.2336 × 10^−4^	2.2184 × 10^−4^	2.2437 × 10^−4^
Yaw (°)	1–100 s	Mean	0.1052	0.2550	0.4849	0.0681
		STD	2.0690	2.7473	2.9716	1.4314
	101–200 s	Mean	0.0348	0.0350	0.0345	0.0303
		STD	0.0822	0.0645	8.7310 × 10^−3^	1.1250 × 10^−3^

**Table 5:** Error statistics of the OPREQ and OBA algorithms.

Items			OBA	OPREQ
Pitch (°)	1–100 s	Mean	2.8284 × 10^−3^	2.8185 × 10^−3^
		STD	2.1098 × 10^−4^	2.1242 × 10^−4^
	101–200 s	Mean	2.8385 × 10^−3^	2.8514 × 10^−3^
		STD	2.0841 × 10^−4^	2.0566 × 10^−4^
Roll (°)	1–100 s	Mean	−2.7945 × 10^−3^	−2.8051 × 10^−3^
		STD	2.3130 × 10^−4^	2.4626 × 10^−4^
	101–200 s	Mean	−2.7622 × 10^−3^	−2.7169 × 10^−3^
		STD	2.2221 × 10^−4^	2.2437 × 10^−4^
Yaw (°)	1–100 s	Mean	0.0645	0.0681
		STD	1.5307	1.4314
	101–200 s	Mean	0.0320	0.0303
		STD	4.0898 × 10^−3^	1.1250 × 10^−3^

**Table 6:** The parameters of the inertial sensors.

Parameters	Gyroscope	Accelerometer
Measurement range	−300~+300°/s	−20~+20 g
Repetitiveness-of-scale factor	≤50 ppm (1σ)	$<3.5\times 10^{-5}\;\left(1\sigma \right)$
Constant bias	<0.02°/h(1σ)	$<5\times 10^{-3}\;g\;\left(1\sigma \right)$
Random bias	$<0.005{}^{\circ}/\sqrt{h}$	$<5\times 10^{-3}\;g\;\left(1\sigma \right)$

**Table 7:** The parameters of the swing model in the turntable test.

Items	Pitch (θ)	Roll (γ)	Yaw (ψ)
Amplitude (°)	3	3	2
Frequency (Hz)	0.15	0.2	0.125
Initial phase (°)	0	0	0
Swaying center (°)	2	−2	135

**Table 8:** The error statistics of the REQUEST and OPREQ algorithms.

Items			ρ=0.1	ρ=0.01	ρ=0.001	Optimal ρ
Pitch (°)	101–200 s	Mean	−0.0162	−0.0163	−0.0176	−0.0167
		STD	6.8578 × 10^−3^	4.6358 × 10^−3^	4.6175 × 10^−3^	4.6189 × 10^−3^
	201–300 s	Mean	−0.0160	−0.0161	−0.0172	−0.0166
		STD	7.0828 × 10^−3^	4.1655 × 10^−3^	4.1284 × 10^−3^	4.1243 × 10^−3^
Roll (°)	101–200 s	Mean	−2.3349 × 10^−3^	−2.3849 × 10^−3^	−4.1816 × 10^−3^	−3.0617 × 10^−3^
		STD	8.9051 × 10^−3^	6.1719 × 10^−3^	6.1717 × 10^−3^	6.1656 × 10^−3^
	201–300 s	Mean	−2.4220 × 10^−3^	−2.5351 × 10^−3^	−3.9452 × 10^−3^	−3.3058 × 10^−3^
		STD	8.5237 × 10^−3^	5.3127 × 10^−3^	5.3100 × 10^−3^	5.3035 × 10^−3^
Yaw (°)	101–200 s	Mean	0.0233	0.0141	−0.3962	−0.1304
		STD	1.3732	0.0399	0.1262	0.0551
	201–300 s	Mean	0.0903	0.0724	−0.1262	−0.0218
		STD	0.8655	0.0219	0.0471	0.0183

**Table 9:** The error statistics of the OBA and OPREQ algorithms.

Items			OBA	OPREQ
Pitch (°)	101–200 s	Mean	−0.0164	−0.0167
		STD	4.6734 × 10^−3^	4.6189 × 10^−3^
	201–300 s	Mean	−0.0163	−0.0166
		STD	4.1922 × 10^−3^	4.1243 × 10^−3^
Roll (°)	101–200 s	Mean	−2.4783 × 10^−3^	−3.0617 × 10^−3^
		STD	6.2884 × 10^−3^	6.1656 × 10^−3^
	201–300 s	Mean	−2.6563 × 10^−3^	−3.3058 × 10^−3^
		STD	5.3849 × 10^−3^	5.3035 × 10^−3^
Yaw (°)	101–200 s	Mean	−0.0200	−0.1304
		STD	0.0699	0.0551
	201–300 s	Mean	0.0467	−0.0219
		STD	0.0263	0.0183
